# Opportunistic bacteria with reduced genomes are effective competitors for organic nitrogen compounds in coastal dinoflagellate blooms

**DOI:** 10.1186/s40168-021-01022-z

**Published:** 2021-03-24

**Authors:** Yu Han, Nianzhi Jiao, Yao Zhang, Fan Zhang, Chen He, Xuejiao Liang, Ruanhong Cai, Quan Shi, Kai Tang

**Affiliations:** 1 State Key Laboratory of Marine Environmental Science, Fujian Key Laboratory of Marine Carbon Sequestration, College of Ocean and Earth Sciences, Xiamen University, Xiamen, 361102 People’s Republic of China; 2 Department of Molecular Virology & Microbiology, Center for Metagenomics and Microbiome Research, Baylor College of Medicine, Houston, TX 77030 USA; 3 State Key Laboratory of Heavy Oil Processing, China University of Petroleum (Beijing), Beijing, 102249 People’s Republic of China

**Keywords:** Dissolved organic matter, Reduced genome, Bacteria, Nitrogen, Blooms

## Abstract

**Background:**

Phytoplankton blooms are frequent events in coastal areas and increase the production of organic matter that initially shapes the growth of opportunistic heterotrophic bacteria. However, it is unclear how these opportunists are involved in the transformation of dissolved organic matter (DOM) when blooms occur and the subsequent impacts on biogeochemical cycles.

**Results:**

We used a combination of genomic, proteomic, and metabolomic approaches to study bacterial diversity, genome traits, and metabolic responses to assess the source and lability of DOM in a spring coastal bloom of *Akashiwo sanguinea*. We identified molecules that significantly increased during bloom development, predominantly belonging to amino acids, dipeptides, lipids, nucleotides, and nucleosides. The opportunistic members of the bacterial genera *Polaribacter, Lentibacter,* and *Litoricola* represented a significant proportion of the free-living and particle-associated bacterial assemblages during the stationary phase of the bloom. *Polaribacter marinivivus*, *Lentibacter algarum*, and *Litoricola marina* were isolated and their genomes exhibited streamlining characterized by small genome size and low GC content and non-coding densities, as well as a smaller number of transporters and peptidases compared to closely related species. However, the core proteomes identified house-keeping functions, such as various substrate transporters, peptidases, motility, chemotaxis, and antioxidants, in response to bloom-derived DOM. We observed a unique metabolic signature for the three species in the utilization of multiple dissolved organic nitrogen compounds. The metabolomic data showed that amino acids and dipeptides (such as isoleucine and proline) were preferentially taken up by *P. marinivivus* and *L. algarum*, whereas nucleotides and nucleosides (such as adenosine and purine) were preferentially selected by *L. marina*.

**Conclusions:**

The results suggest that the enriched DOM in stationary phase of phytoplankton bloom is a result of ammonium depletion. This environment drives genomic streamlining of opportunistic bacteria to exploit their preferred nitrogen-containing compounds and maintain nutrient cycling.

Video abstract

**Supplementary Information:**

The online version contains supplementary material available at 10.1186/s40168-021-01022-z.

## Background

Phytoplankton and heterotrophic bacteria, together, play a central role in the oceanic carbon cycles [1, 2]. Heterotrophic bacteria interact with the phytoplankton community via utilization of phytoplankton-produced organic matter for biomass production and respiration [3]. Therefore, the community structure of phytoplankton affects the diversity of bacteria community [4–6]. On the other hand, interactions among microbes are critical in controlling the diversity, dynamics, and fates of phytoplankton in the forms of labile organic matter recycling and cross feeding of nutrients, vitamins, and signaling molecules [1, 7, 8]. These interactions between bacteria and phytoplankton are heavily dependent on the distribution of organic matter and nutrients [9–11].

Heterotrophic bacteria can be classified as either oligotrophic or opportunist [12]. Oligotrophs are generally found in environments with low levels of nutrients. Opportunistic bacteria can exploit high nutrient niches with a broad spectrum of substrates [12, 13]. Phytoplankton blooms provide an ideal scenario to study the responses of opportunists to transient organic matter and nutrient pulses and examine their functional roles in ecosystems. Previous reports on opportunists in blooms have focused on dynamics of the bacterial community and their metabolic functions by meta-omics or the interactions between bacteria with phytoplankton through co-cultivation [5, 7, 14–18]. Known opportunitrophs, members of *Bacteroidetes*, *Alphaproteobacteria*, and *Gammaproteobacteria*, are abundant within blooms situated in the cold and temperate waters of the open ocean and coastal zones, and are reported to possess broad genomic and metabolic repertoires that allow them to exploit various substrates (e.g., carbohydrates, proteins, lipids) as energy sources [4, 17]. On the other hand, opportunitrophs are also reported have a complimentary metabolic strategies that enabled them to co-thrive in the bloom [19]. *Bacteroidetes* have been shown to decompose high-molecular weight macro-molecules (e.g., polysaccharide-like and protein-like substances) into lower weight molecules (< 1000 Da) in blooms, and members of the *Alphaproteobacteria* and *Gammaproteobacteria* can transform and mineralize low weight organic matter [17, 19]. Although they appear to be the dominant groups in blooms, few studies have identified common characteristics that give them a competitive advantage. “Meta-omics” technologies, such as metagenomics, metatranscriptomics, and metaproteomics, have measured some metabolic potential of marine heterotrophic bacteria [20–22]. Recent studies have reported bacterial community structures are shifted by the components and partitioning of labile dissolved organic matter (DOM) resource [18, 23]. However, few assessments linking bacterial community composition and organic matter lability during blooms have been made.

In this study, we focused on opportunist bacteria incubated with bloom DOM to determine the metabolic value and lability of organic compounds. Our combined data of field observations and lab-based cultivation indicated that opportunists differ substantially in their genomic features and ability to utilize organic matter, providing them a competitive advantage during phytoplankton blooms.

## Results and discussion

### Environmental conditions and sampling sites characteristics

A dinoflagellate (*Akashiwo sanguinea*) bloom occurred in Wuyuan Bay, Xiamen, China (24.58° N, 118.18° E), during February and March 2016 (Additional file 1: Figure S1A, B). *A. sanguinea* blooms often occur in coastal areas and can lead to mass mortality of invertebrates and fish [24–30]. Chlorophyll a (Chl *a*) and dissolved oxygen (DO) significantly increased during the exponential phase of the bloom, and the stationary phase lasted for 20 days (Additional file 1: Figure S1C). Samples were taken approximately 1–3 km offshore from Xiamen City at central (C) (24.59° N, 118.16° E) and peripheral (P) (24.54° N, 118.20° E) sites (Additional file 1: Figure S1B). There was a higher Chl *a* concentration at the C site than the P site (Table 1). During the early- (stage I) and mid- (stage II) stationary phase, the levels of nitrate, nitrite, and phosphate decreased with the development of oxygenic photosynthetic process, as indicated by the increased Chl *a*, pH, and DO, suggesting that inorganic nutrients were consumed by phytoplankton in the bloom. However, ammonium increased at the mid-stationary phase, reaching approximately 7 μmol l^−1^ at site P. Dissolved organic carbon (DOC) increased by nearly 3-fold during early-stationary phase as compared to mid-stationary phase (Table 1), showing that bloom progression affected organic matter dynamics. The total prokaryotic abundance at site P increased by 2.5-fold during mid-stationary phase compared to early-stationary phase, while prokaryotic abundance at site C remained unchanged (Table 1).

**Table 1:** Environmental parameters. The samples observed at the center (C) and the peripheral (P) sites during early- (stage I) and mid- (stage II) stationary phase of the bloom. Abbreviations: *Temp*, temperature; *DO*, dissolved oxygen; *Chl a*, chlorophyll a; *DOC*, dissolved organic carbon; *DON*, dissolved organic nitrogen; *TPA*, total prokaryotic abundance; *N.D.*, not detectable

Samples	Temp (°C)	Salinity	pH	DO (mg l^−1^)	Chl *a* (μg l^−1^)	Nitrate (μmol l^−1^)	Nitrite (μmol l^−1^)	Ammonium (μmol l^−1^)	Phosphate (μmol l^−1^)	Silicate (μmol l^−1^)	DOC (μmol l^−1^)	TPA (10^6^ cells ml^−1^)
P (I)	14.16	27.37	8.32	9.56	13.65	42.26	2.61	N.D.	1.31	36.23	75.72 ± 2.38	1.70 ± 0.26
C (I)	14.98	25.15	8.70	11.20	145.40	55.64	4.19	N.D.	1.87	45.55	221.73 ± 38.93	4.94 ± 0.15
P (II)	14.90	27.94	8.65	11.88	45.31	8.35	0.65	7.34	0.02	19.77	186.39 ± 5.11	4.20 ± 0.36
C (II)	16.80	25.41	8.77	13.63	152.10	9.36	1.71	0.14	0.20	46.12	689.12 ± 28.14	4.34 ± 0.15

### Characterization of in situ DOM by untargeted analysis

**Fig. 1:**
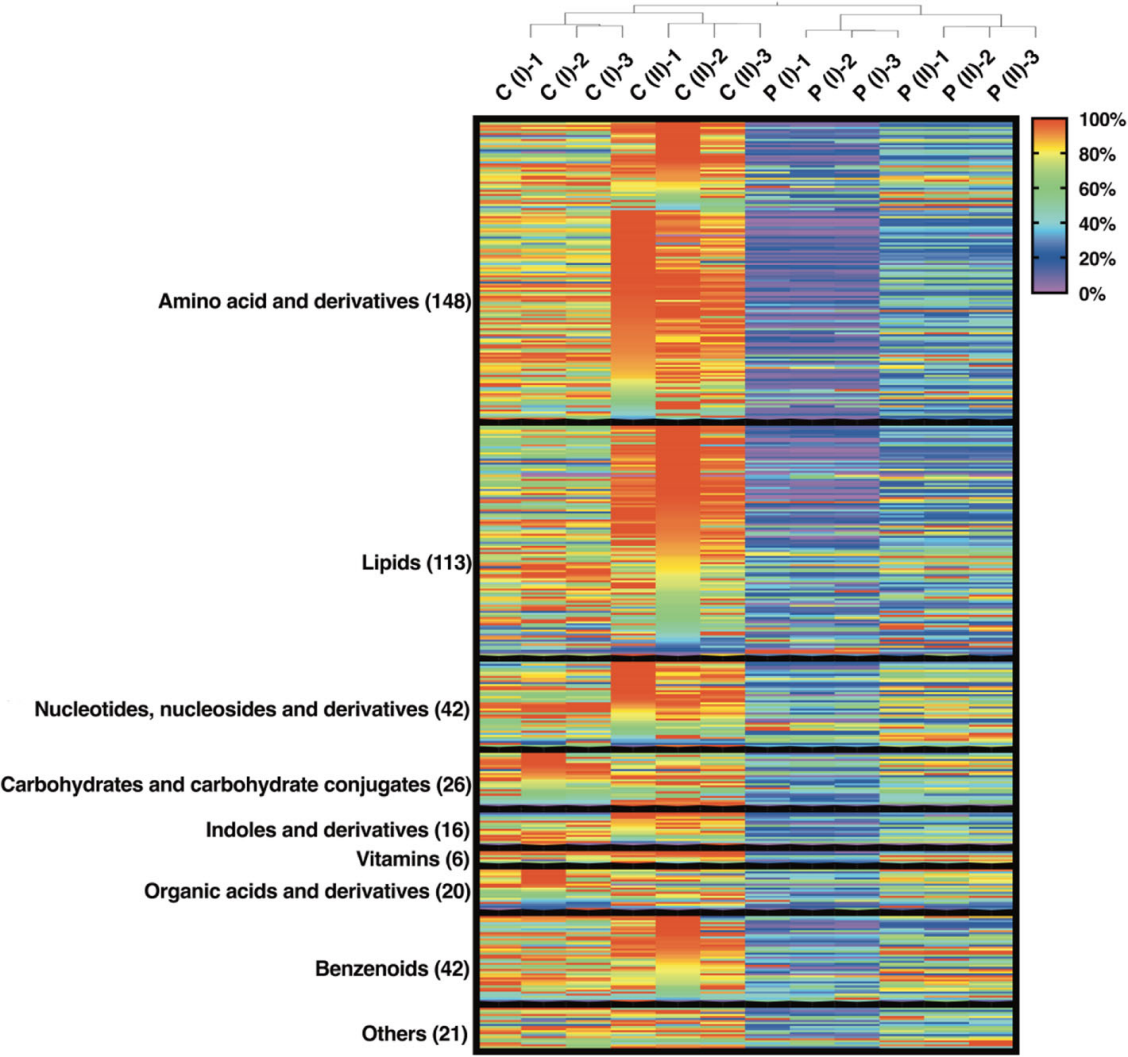
Characterization of dissolved organic matter components in blooms. Metabolite patterns (434 compounds displayed as the peak area) observed at the center (C) and peripheral (P) sites during early- (stage I) and mid- (stage II) stationary phase of the bloom. Each compound is normalized across each row to a range between 0 and 100%, with 100% representing the highest relative proportion between samples. Metabolites are categorized into nine chemical classes and the number of compounds are given in the parentheses. Sample replicates (*n* = 3) of each condition are presented

A total of 434 compounds, comprising a broad range of metabolites, were identified in the bloom. These included amino acids and their derivatives (including dipeptides), lipids, nucleotides and nucleosides, carbohydrates and their derivatives, indoles, organic acids, and benzenoids (Fig. 1). Compound details are presented in Additional file 2: Table S1. Approximately 92% of the total detected compounds were found at a higher abundance in site C compared to site P and approximately 85% of the total detected compounds were found at higher abundances at during stage II than at stage I. The compounds found to be increasing were mainly amino acids, peptides, lipids, nucleotides, and nucleosides, accounting for more than 70% of the total detected compounds (Fig. 1). Lipids were are enriched in *A. sanguinea* and comprised up to 40% of the dry cell weight [31]. The increased abundance of metabolites correlated positively (correlation *R* = 0.77, *p* = 0.002, Mantel Test) with the increased DOM in the bloom, suggesting that phytoplankton contributed to the DOM pool. Previous research on blooms has primarily focused on the microbial community, potential functions, and some chemical signals [7], while only a few studies have been conducted on the composition of DOM, whereas phytoplankton are a major dissolved organic nitrogen (DON) source [32, 33]. Cultivated marine *Synechococcus* is reported to contribute a variety of nitrogen-rich compounds to DOM, including amino acids, oligopeptides as well as nucleosides, and indoles [34, 35]. Geider et al. [36] found cyanobacteria and diatoms contain 30–60% proteins by dry weight, which are the largest compounds; however, dinoflagellates released more nitrogen-containing substances, while diatoms also released a considerable amount of carbohydrates such as polysaccharides. It was found that DON substances, such as amino acids, peptides, nucleotides, and nucleosides, are significant components of bloom seawater, and these substances may play an important role in shaping microbial community structure and allow opportunists to flourish.

### Bacterial community composition succession during the bloom

**Fig. 2:**
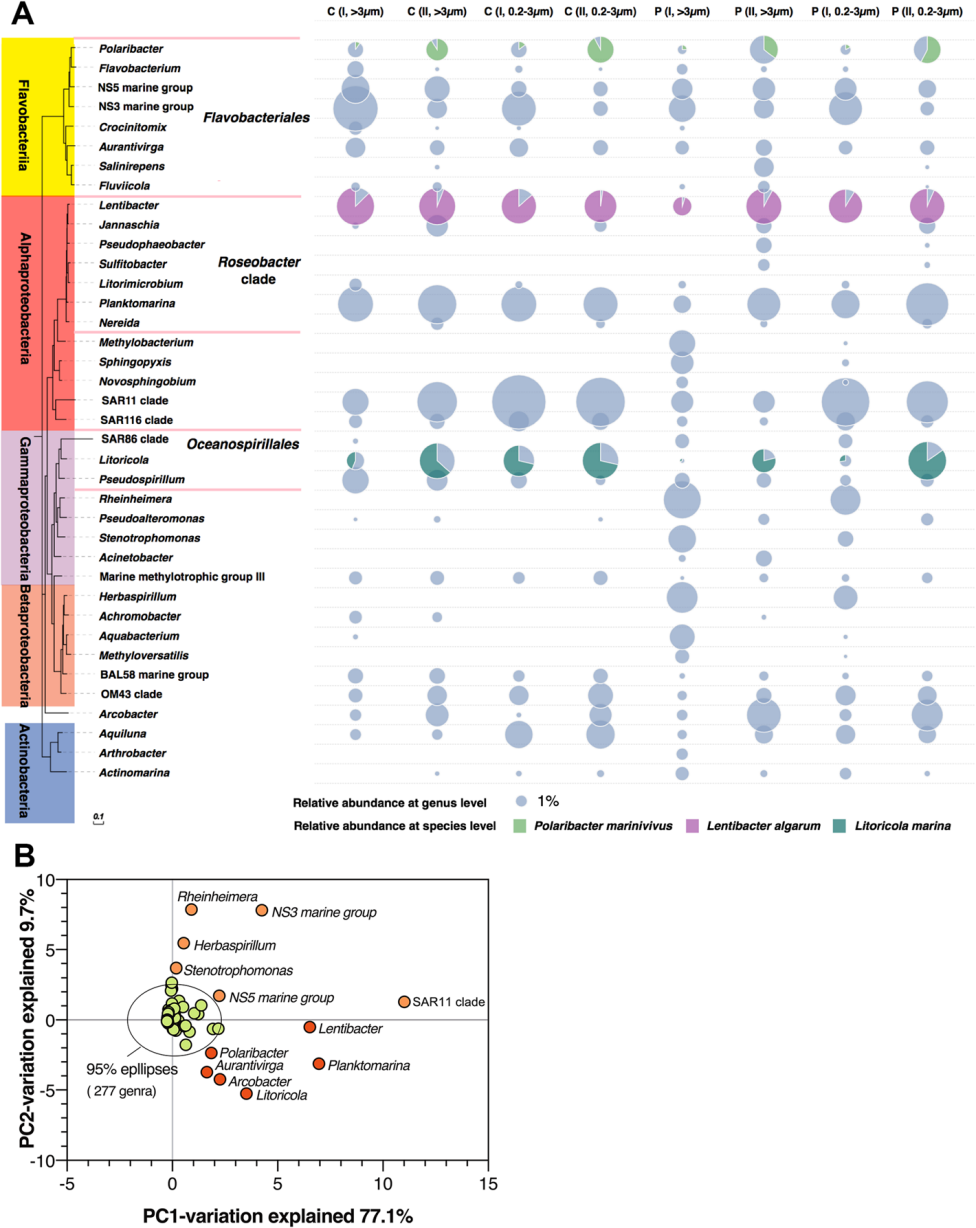
Dynamics of bacterial groups measured by the 16S rRNA gene sequences in blooms. The results show the dominant group change in early- (stage I) and mid- (stage II) stationary phase of the bloom at the center (C) and peripheral (P) sites with particle-associated (> 3 μm) or the free-living (0.2–3 μm) strategies (**a**). The bacteria belonging to the six most abundant classes are shown in the phylogenetic tree. The taxonomic information is the name associated with their best sequence match within the EZbiocloud database (see the “Methods” section). The size of the gray solid circle indicates the relative abundance of each operational taxonomic unit at the genus level. The grass green, rosy, and indigo sections indicate the proportion of species within their corresponding genus. Principal component analysis of bacterial communities (289 genera) between stage I with stage II were divide into three categories (**b**). Twelve genera with decreased (orange solid circle) or increased (red solid circle) relative abundance represent relatively greater changes in the relative abundances compared to others (green circles enclosed by the ellipse)

The organisms detected in the bloom belonged to 24 bacterial classes from 11 phyla (Additional file 3: Table S2). The majority were *Alphaproteobacteria* (29–55%), *Gammaproteobacteria* (13–25%), and *Flavobacteria* (16–35%) across all particle-associated and free-living samples. *Betaproteobacteria* and *Actinobacteria* accounted for a smaller proportion (~ 9 % and ~ 3%, respectively, Fig. 2a). The number of genera was greatest within the *Alphaproteobacteria* (80 genera), *Gammaproteobacteria* (66 genera), and *Flavobacteriia* (39 genera) (Additional file 3: Table S2). The most abundant bacteria in the coastal bloom included *Roseobacter* clade: *Lentibacter* (3–11%), *Planktomarina* (3–14%); *Flavobacteriales*: *Polaribacter* (1–6%), NS5 marine group (2–6%), NS3 marine group (2–16%); *Oceanospirllales*: *Litoricola* (1–10%), and SAR11 clade (4–25%).

Microbial community composition at the genus level was significantly different (*q* < 0.05, analysis of similarities (ANOSIMS)) between stage I and stage II. Principal component analysis of stage I and stage II bacterial communities showed a clear separation between genera that decreased and increased in relative abundance, which suggested shifts in microbial community composition during the bloom (Fig. 2b). Twelve genera with high PC1 or PC2 scores represented relatively greater changes in relative abundances as compared to others (enclosed by the ellipse in Fig. 2b). Of these genera, SAR11 was the most abundant in all samples, but they decreased significantly during stage II (*q* < 0.05, ANOSIMS). Similarly, a sharp decrease in NS5 and NS3 marine groups, the most common *Flavobacteria* taxa, was observed. They accounted for 3–11% of the relative abundance of the bacterial community at stage I but decreased to 2–4% by stage II (Fig. 2a). *Rheinheimera*, *Stenotrophomonas,* and *Herbaspirillum* were only observed during stage I at the P site and together accounted for 25% and 15% of the relative abundance of particle-associated and free-living bacterial communities, respectively. In contrast, regardless of the particle-associated and free-living size fractions, the relative abundance of bacteria belonging to *Polaribacter*, *Lentibacter*, *Planktomarina,* and *Litoricola* increased significantly (*q* < 0.05) during stage II, suggesting that they can respond to pulses of newly available resources and outcompete other taxa that are initially more abundant. At the species level, *Polaribacter marinivivus*, *Lentibacter algarum, Planktomarina temperata,* and *Litoricola marina*, which accounted for 6–74%, 68–82%, 46–80%, and 53–67% of their respective genera (Fig. 2a), were the dominant species in the bloom.

A similar limited number of heterotrophic bacterial lineages, members of *Flavobacteriales*, *Roseobacter* clade, and *Ocenospirllales,* have been observed in various algal blooms caused by dinoflagellates, diatoms, or cyanobacteria [4, 7, 17, 37–39]. In addition, *Polaribacter* has been shown to distinctly respond to bloom [7, 17, 18], and the first isolates of *Lentibacter algarum*, *Planktomarina temperata*, and *Litoricola marina* were isolated and identified from blooms [21, 40, 41]. However, our study identified several novel taxonomic signatures at the species level, which were distinctly abundant in this bloom, such as *Polaribacter marinivivus*, *Lentibacter algarum*, *Planktomarina temperata*, and *Litoricola marina*. This provided a selective and representative group of strains for studying the competitive advantages of dominant bacteria in blooms.

### Cultivable bacteria in the bloom

A total of 216 isolates were assigned to *Alphaproteobacteria* (81 strains), *Gammaproteobacteria* (74 strains), *Flavobacteriia* (15 strains), *Bacilli* (40 strains), and *Actinobacteria* (5 strains) (Additional file 4: Table S3). According to the abundance of the microbial community in the bloom, we isolated the dominant strains, *Polaribacter marinivivus* LXJ4 (CGMCC 1.16109), *Lentibacter algarum* HYO3 (CGMCC 1.15876), and *Litoricola marina* HY2016 (CGMCC 1.17774), as representatives for further analysis of metabolic mechanisms. The abundance and taxonomy in *Planktomarina temperata* are similar to *Lentibacter algarum*, and its related functional genes have been studied for reference in this study [21]. Interesting, of those isolates, only a few belonged to the dominant groups*,* including *Lentibacter* (5 strains), *Polaribacter* (2 strains), and *Litoricola* (1 strain); no strains of *Planktomarina* were isolated. This result was contrary to their abundance in the microbial community of the bloom, causing us to speculate that the culture medium possibly suppressed the dominant bacteria. However, the cultured bacterial isolates showed the dominance of *Gammaproteobacteria*, especially *Pseudoalteromonas*, organisms known to be rapidly growing opportunistic microorganisms in resource-rich media (Additional file 4: Table S3). Also, *Pseudoalteromonas* is often observed in coastal seawater [22, 23, 42], but the proportion during the stationary phase of the bloom was only 0.4%. *Pseudoalteromonas* is often associated with the decline of the bloom when dead and lytic phytoplankton cells release larger amounts of DOM compared to other bloom stages [22]. The results suggested that despite the rise of DOM in the bloom to support bacterial growth, there may still be some nutritional restrictions, as only few distinct groups expanded rapidly.

### Genome streamlining of species

**Fig. 3:**
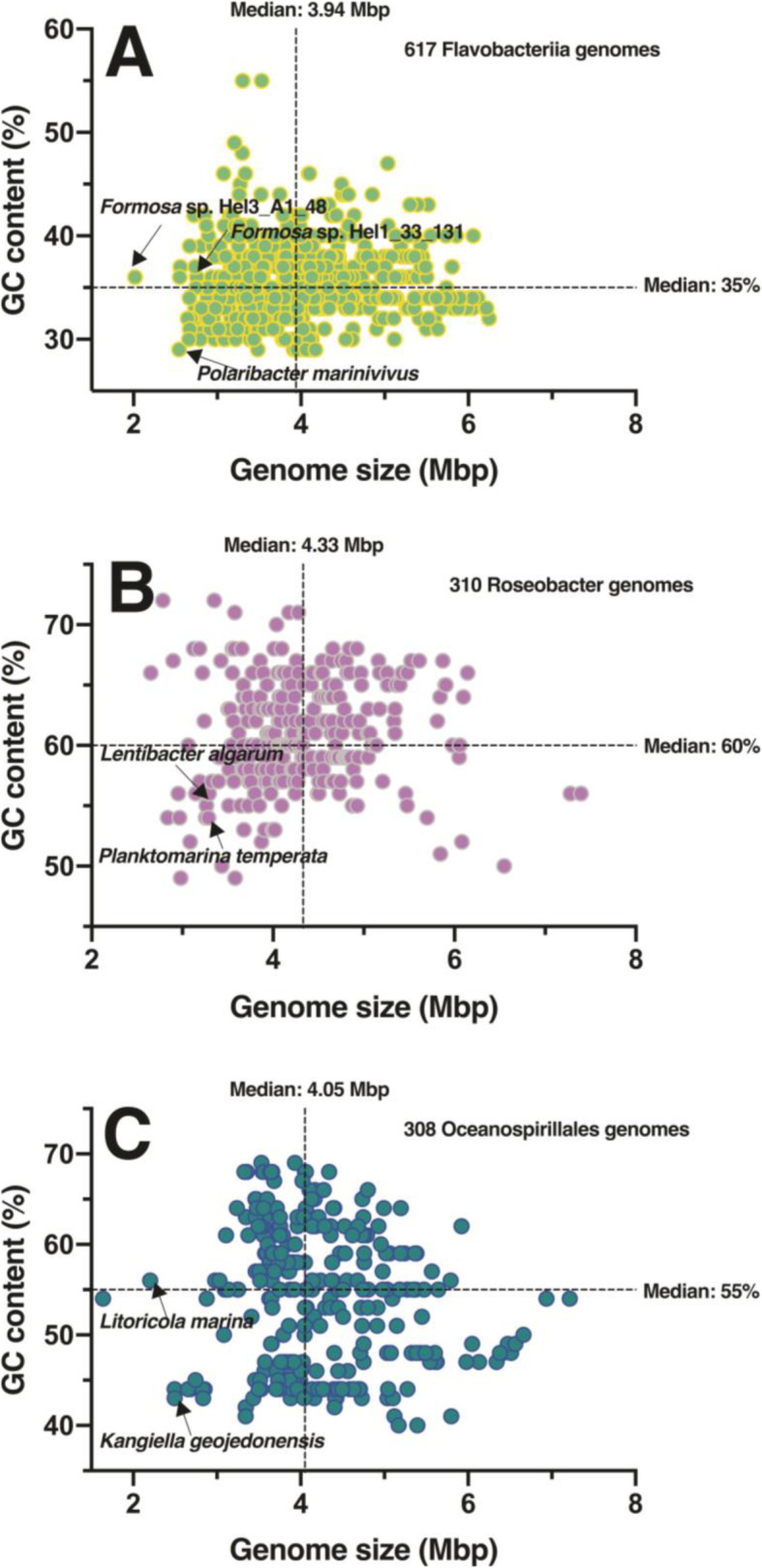
Genome traits on three representative isolates in comparison to reference strains. Genomic information of reference strains belong to the homologous class level of contained *Flavobacteria* with grass green (**a**), *Roseobacter* with rosy (**b**), and *Oceanospirillales* with indigo (**c**) downloaded from the Integrated Microbial Genomics (IMG) site (http://img.jgi.doe.gov). Each solid colored circle represents a strain, and the dotted lines represent the median of genome size and GC content. The arrows indicate the reference strains with genome streamlining reported previously or the isolates in this study

Within bloom bacterial communities, SAR11 clade genomes are highly streamlined [43–45], and *Planktomarina temperata* also shows features of genome streamlining such as the reduction and optimization of the genetic material [21]. *P. marinivivus*, *L. algarum*, and *L. marina* also exhibited features of genome streamlining (Fig. 3) as characterized by small genome size and low GC content and non-coding densities [46]. The genome size of *L. algarum* (3.29 Mbp) was similar to *Planktomarina temperata* and was located in the low GC content area (< 60%) for *Roseobacter* genomes (Fig. 3b). In addition, plasmids, clustered regularly interspaced short palindromic repeats (CRISPR), and complete gene transfer agents that are typical genetic features of many *Roseobacter* were not found in the *L. marina* genome [21, 47, 48]. Similarly, the GC content (29%) of *P. marinivivus* was the lowest among the current sequenced marine *Flavobacteriia*, and the genome size (2.56 Mbp) was only larger than *Formosa* sp. Hel3_A1_48 and smaller than the genomically streamlined *Formosa* sp. Hel1_33_131, both of which have been found associated with diatom blooms [20]. *L. marina* had the second smallest genome size (2.2 Mbp) among the *Oceanospirillaceae* strains, and its GC content had a median value of 55%. Compared with existing relatives, the *P. marinivivus*, *L. algarum,* and *L. marina* chromosomes had reduced levels of non-coding DNA, harboring a total of 2419, 3262, and 2311 predicted protein-coding sequences, respectively (Additional file 5: Table S4).

Considering the heterotrophic ability of these strains, an intriguing genome feature was the presence of multiple transporters for various organic nitrogen molecules, peptidases for protein and peptide degradation, and glycoside hydrolase (GH) for the hydrolysis of glyosidic bonds in organic matter. The highest number of transporter genes was found in *L. algarum* (291 genes), followed by *L. marina* (203 genes) and *P. marinivivus* (137 genes). Genes encoding the ATP-binding cassette (ABC) transporters were common in *L. algarum* (181 genes) and *L. marina* (125 genes), while genes encoding TonB-dependent transporters were found only in *P. marinivivus* (41 genes). *P. marinivivus*, *L. algarum*, and *L. marina* had 65 (29 superfamilies), 55 (32 superfamilies), and 26 (18 superfamilies) genes encoding peptidases, respectively. GH genes were more abundant in *P. marinivivus* (33 genes, 17 superfamilies) than *L. algarum* (10 genes, 5 superfamilies) and *L. marina* (6 genes, 3 superfamilies) (Additional file 6: Table S5). Although the small genome size can possibly be attributed to gene loss, they still encoded essential metabolic functions. *P. marinivivus* contained fewer genes encoding peptidases and GH than *Polaribacter* sp. Hel1_33_49 and *Polaribacter* sp. Hel_I_85 that were isolated from a diatom-dominated bloom [20], but their functional superfamilies were similar (Additional file 6: Table S5). This suggested that encoding genes were diminished, but not functionally absent. Similar to *Planktomarina temperata* [21], the total number of transporters in *L. algarum* was less than the median value of 380 in *Roseobacter*. There were few ABC transporter genes for the uptake of carbohydrates (5 genes), but genes for the uptake of amino acids (28 genes), peptides (31 genes), and polyamines (like putrescine and spermidine, 38 genes) were enriched in the *L. algarum* genome. *L. marina* lacked genes for hydrocarbon utilization and collagen degradation that were identified in *Oceanospirillaceae* strains from oil spill and eukaryotic endosymbionts, respectively. Compared to the known genome-reduced *Kangiella* strains belonging to *Oceanospirillaceae* [49], *L. marina* had a higher number of genes assigned to the clusters of orthologous genes (COGs) “amino acid transport and metabolism,” “carbohydrate transport and metabolism,” and “nucleotide transport and metabolism”.

Surprisingly, the isolates in this study encoded for a greater number of genes related to the transport of DON, especially in amino acids, peptides, and nucleotides, relative to the other non-streamlined strains. The potential to transport various nitrogen-containing substrates were compared with the reference strains, belonging to the corresponding class level, detailed in Additional file 5: Table S4. For example, we compared three non-streamlined strains, which had low abundance in the bloom, *Dokdonia donghaensis* MED134 (*Flavobacteria*), *Roseobacter* sp. MED193 (*Roseobacter*), and *Neptuniibacter caesariensis* MED92 (*Oceanospirillales*), for one-to-one correspondence [50]. The results showed that the *P. marinivivus* possessed the additional ability to transport proline, threonine, and vitamin B12 compared to *D. donghaensis* MED134. Three additional genes for amino acid transporters (histidine, lysine, and threonine) were encoded in* L. algarum* relative to *R.* sp. MED193. *L. marina* had additional transporters for arginine, cysteine, lysine, threonine, nucleobase, nucleoside, purine, and vitamin B12, in comparison to *N. caesariensis* MED92 (Additional file 5: Table S4). More interestingly, the isolates also had more abundant DON transporters compared to the aforementioned streamlined bacteria (e.g., *Formosa* sp. Hel3_A1_48, *Planktomarina temperata* RCA23, and *Kangiella geojedonensis* KCTC 23420, see details in Additional file 5: Table S4), implying that the streamlined bacteria in the bloom may have had greater potential for DON uptake than other organisms. The genomic results suggested that the genes were reduced, but not functionally absent in the dominant isolates, and the ability to transport and utilize DON, especially amino acids, peptides, and nucleotides, was retained.

### DOM-induced bacterial growth

**Fig. 4:**
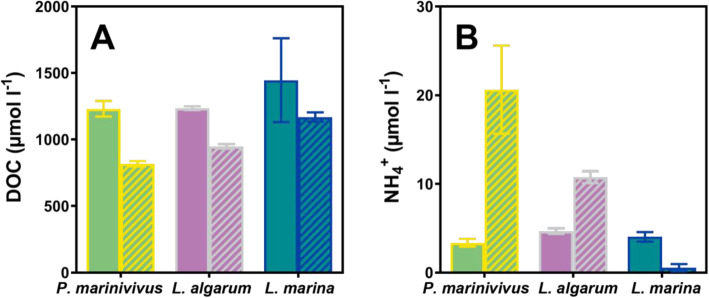
Variation in extracellular concentration of dissolved organic carbon (**a**) and ammonium (**b**) during growth. The samples analyzed in the uninoculated medium (color-filled) and the end of cultivation (color-filled with diagonal lines). The bars with grass green, rosy, and indigo represent *P. marinivivus* LXJ4, *L. algarum* HYO3, and *L. marina* HY2016, respectively. The average value of triplicate cultures (*n* = 3) is shown (error bars represent one standard deviation)

*P. marinivivus*, *L. algarum,* and *L. marina* all grew rapidly in response to an impulse of bloom DOM. Their average growth rates were 0.35 ± 0.03 h^−1^, 0.47 ± 0.05 h^−1^, and 0.79 ± 0.21 h^−1^, respectively (Additional file 1: Figure S2). All DOM amendments resulted in increased bacterial abundances (up to 5–6 × 10^6^ cell ml^−1^) and declines in DOC concentrations. In a *P. marinivivus* incubation experiment, DOC declined an average of 413 μmol l^−1^ by the end of the stationary phase, accounting for 34% of the total DOC. The decline of DOC in *L. algarum* and *L. marina* experiments accounted for 23% and 19% of the total DOC, respectively (Fig. 4a). The extracellular ammonium concentrations in the *P. marinivivus* and *L. algarum* incubations increased on average by 17 μmol l^−1^ and 6 μmol l^−1^, respectively. However, ammonium in *L. marina* decreased on average by 3 μmol l^−1^ (Fig. 4b).

### Proteomic responses of isolates to DOM amendments

**Fig. 5:**
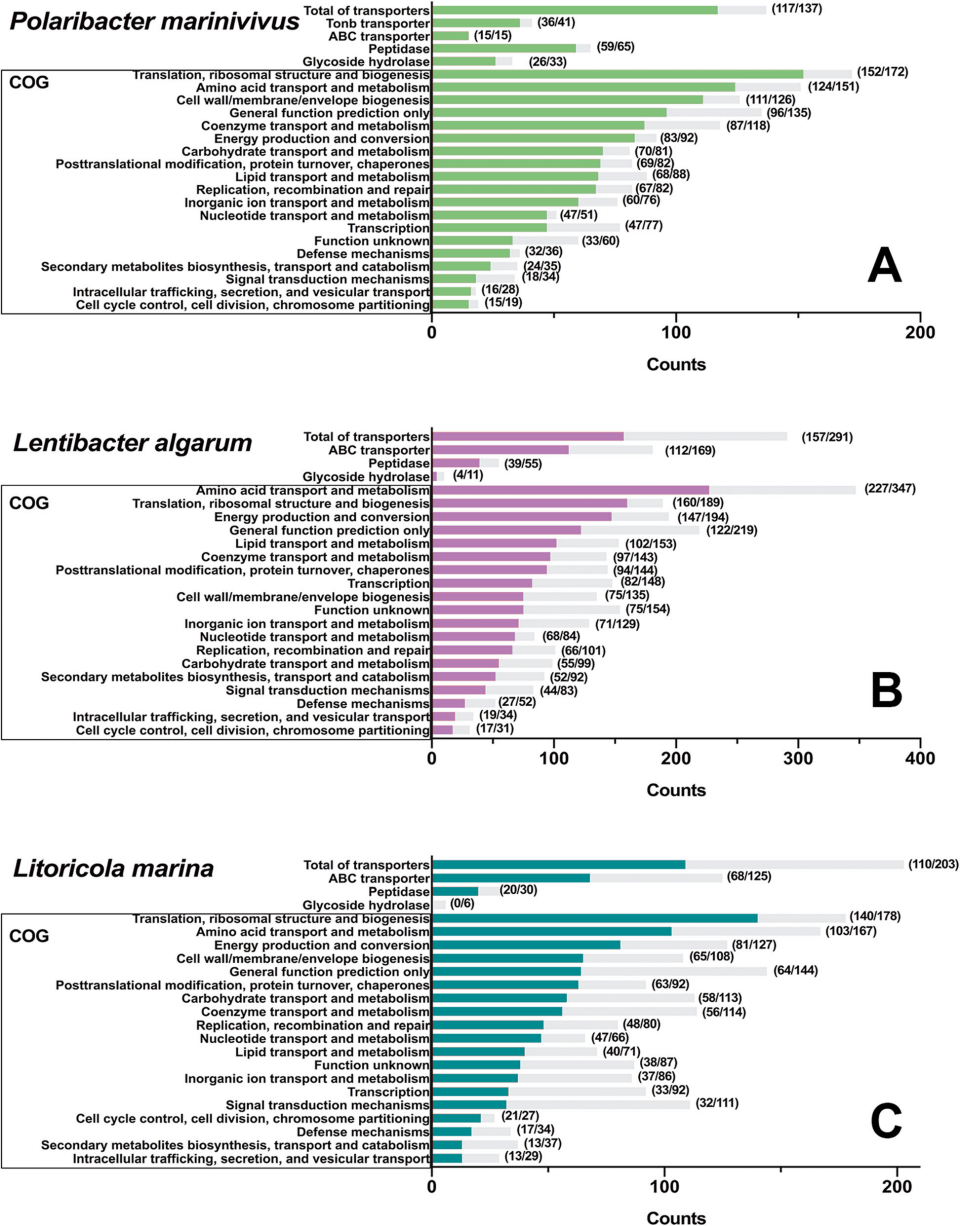
Proteomic responses of three isolates during growth. *P. marinivivus* LXJ4 (**a**), *L. algarum* HYO3 (**b**), and *L. marina* HY2016 (**c**) were cultivated in autochthonous seawater from bloom. Differential protein expression counts (grass green, rosy or indigo) and gene counts (gray) of transporters, peptidase, glycoside hydrolase, and clusters of orthologous gene (COG) functions in three isolates are shown with the specific counts list in parentheses. Sample replicates (*n* = 3) of each condition are presented

A total of 1735, 1722, and 1067 proteins were identified during growth, representing 71.7%, 52.8%, and 46.2% of all predicted proteins encoded in the *P. marinivivus*, *L. algarum*, and *L. marina* genomes, respectively (Additional file 7: Table S6). The proportion of protein expression in DOM amended medium nearly reached the level observed during cultivation in nutrient rich marine broth medium (59–80%) [50]. Proteomic analysis revealed that more than 50% of the predicted genes were expressed in each COG functional group (Fig. 5). Here, we highlight the expressions of transporter, peptidase, GH, polysaccharide utilization loci (PUL), and other essential proteins in the three strains.

#### Transporter

Transporters accounted for ~ 7% of identified protein sequences in *P. marinivivus*, and ∼ 9% and ∼ 10%, respectively, in *L. algarum* and *L. marina*. Furthermore, classic families of the transporter proteins in the three strains were typically located in the high-abundance range, such as TonB-dependent (TBDT) transporters in *P. marinivivus* and ABC transporters in *L. algarum* and *L. marina* (Fig. 5).

TBDT transporters expressed in *P. marinivivus* had predicted substrate specificities to polysaccharide/oligosaccharide, peptide, siderophore, and vitamin B12 (Additional file 7: Table S6). Other transporter genes for the uptake of fatty acids were also expressed (Additional file 1: Figure S3).

Substrate affinities of expressed ABC transporters suggest that amino acids, peptides, polyamines, nucleosides, glycine betaine, and taurine were potential substrates for *L. algarum* (Additional file 1: Figure S3). Transporters targeting fatty acid and carboxylic acid were also identified in *L. algarum* (Additional file 1: Figure S3).

*L. marina* showed the potential ability to take up amino acids, nucleosides, glycine betaine, carbohydrates, phospholipid/fatty acids, carboxylic acid, and thiamine (Additional file 1: Figure S3). Genes encoding nitrate reductase and nitrite reductase involved in nitrate assimilation were only found in the *L. marina* genome, however, they were not expressed (Additional file 7: Table S6). Ammonium transporter was detected in abundance in the *L. marina* proteome, as well as the ammonia assimilation enzyme glutamine synthetase (Additional file 1: Figure S3). This indicated that ammonia from environmental uptake can be incorporated into amino acids. Amino acid transport and metabolism were one of the most common COG functions in the proteome of each isolate (Fig. 5).

#### Peptidase

Only 20 peptidases were detected in *L. marina*. In contrast, 59 and 39 peptidases were detected in *P. marinivivus* and *L. algarum*, respectively (Fig. 5). Most of these scored in the upper third of the distribution, indicating that these functions are overwhelmingly carried out by high-abundance proteins. Compared with *L. algarum* and *L. marina*, *P. marinivivus* expressed more abundant aminopeptidases (16 proteins, e.g., secreted peptidases of M01 family) and carboxypeptidases (10 proteins) that are known to act on polypeptides as nitrogen sources [20] (Additional file 6: Table S5).

#### GH and PUL

Most of the glycoside hydrolases in the *P. marinivivus* genome were expressed (26 proteins). However, only four glycoside hydrolases were identified in *L. algarum*, and none were found in *L. marina* (Fig. 5). In addition, the predicted genes for laminarin and alginate consumption were located in the multi-gene PUL of *P. marinivivus* (Additional file 1: Figure S4). Most of proteins in the two PULs were expressed during the incubation (Additional file 6: Table S5), indicating that *P. marinivivus* has the ability to degrade laminarin and alginate, two major polysaccharides of algae [51].

#### Other essential functions

The expressed proteins involved in motility and chemotaxis in the three isolates provide a mechanism by which microorganisms can respond to microscale DOM gradients and access nutrient-enriched patches [52]. Superoxide dismutase enzymes in *P. marinivivus* and *L. marina* and the peroxiredoxin enzyme in *L. marina* were abundant (Fig. 5) and can serve as antioxidants by scavenging the superoxide radicals and hydrogen peroxide that are produced at an accelerated rate during the bloom [53]. Bacteriorhodopsin is in the upper range of the *P. marinivivus* proteome (Fig. 5), and it might function as a light-driven proton pump enhancing *P. marinivivus* survival via phototrophy [54]. A high concentration of the organosulfur compound dimethylsulfoniopropionate (DMSP) was observed in the bloom (up to 605 nM, Additional file 1: Figure S5) and was likely produced by *A. sanguinea* [55]. The expression of DMSP demethylase showed that *L. algarum* can utilize DMSP as a source of both carbon and sulfur [11] (Fig. 5). These properties may confer competitive advantages to selected bacterial species during blooms.

We found that most of the identified proteins in three isolates were highly expressed, containing an abundance of peptidases and the transport and metabolism of amino acids and nucleotides. The expressed proportion of the proteomes ranged from 7.1 to 13.2% in amino acid transport and metabolism, similar to cultivation under nutrient rich marine broth medium (expressed 7.4–12.1% of the total proteomes with SEED category). However, in carbohydrate transport and metabolism, the expressed proportion of the proteomes was 3.2–5.4%, significantly lower than in rich medium (5.9–8.3%) [50]. In addition, we compared the protein expression of DON transporters, especially in amino acids, peptides, and nucleotides. The isolates cultivated in this study showed a comparable protein functional expression in DON transport to the non-streamlined strains (*D. donghaensis* MED134, *R.* sp. MED193 and *N. caesariensis* MED92) in pervious study using a nutrient rich marine broth medium [50] (Additional file 5: Table S4). In contrast, *Pseudoalteromonadaceae* and *Alteromonadaceae* expressed more TBDT transporters and GH protein to utilize carbohydrates [22].

Combined, the genome and proteome results imply that streamlined isolates with high protein expression during bloom play a significant role in the DON cycle. In detail, *P. marinivivus* expressed more peptidase and *L. algarum* showed high expression of amino acid transport and metabolism, while *L. marina* displayed a preference for the transport and metabolism of amino acids and nucleotides.

### Uptake of DOM components by isolates

**Fig. 6:**
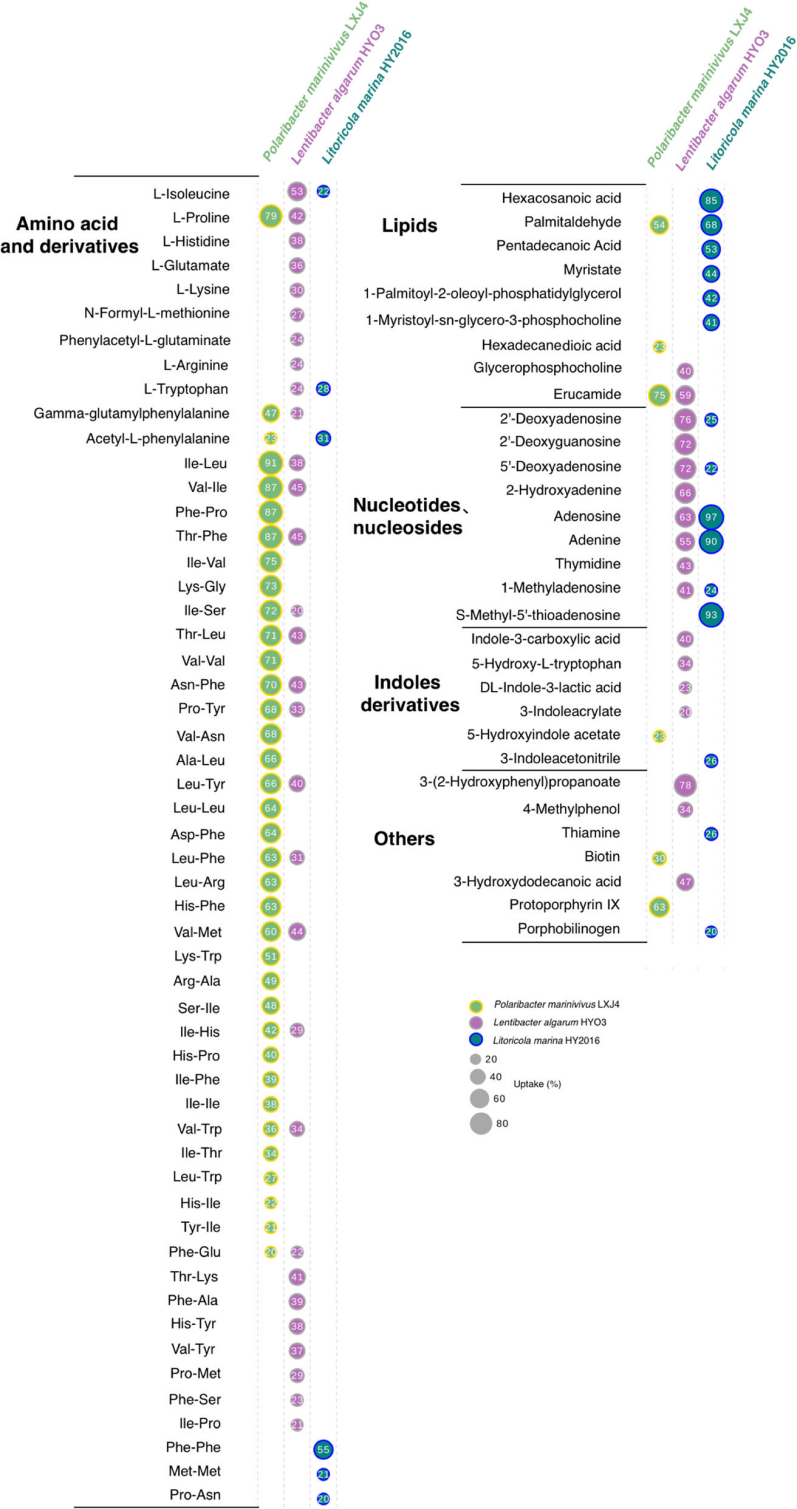
Uptake of specific metabolites of DOM from bloom during growth by three isolates. Metabolites (*n* = 85), mainly containing five classes, are significantly taken up (*p* < 0.05 and relative uptake proportion > 20%) by *P. marinivivus* LXJ4 (grass green), *L. algarum* HYO3 (rosy), and *L. marina* HY2016 (indigo). The size of the circle represents the relative proportion consumed by the species in culture and the specific value inside of circle. Three culture replicates (*n* = 3) of each condition were used

Metabolite uptake was determined as the percentage of a metabolite depleted from the medium, as compared to the uninoculated control medium, at the end of stationary phase (Additional file 8: Table S7). A total of 85 compounds in the medium were significantly decreased (*p* < 0.05, *T*-test) with at least 20% uptake of each metabolite, among them amino acids and their derivatives (including dipeptides), lipids, nucleotides and nucleosides, and indole derivatives. The depletion profiles showed large differences in substrate utilization among the three isolates (Fig. 6). *L. algarum* take up nine free amino acids with 24–53% depletion, whereas *P. marinivivus* take up alanine and proline with 23–79% depletion. A total of 34 kinds of dipeptides, with the majority being depleted by more than 50%, were taken up by *P. marinivivus*, and 21 kinds of dipeptides were taken up by *L. algarum* with 21–45% depletion. *L. marina* only removed five amino acids and dipeptides, but it could take up a long-chain fatty acid and two short-chain fatty acids, two phosplipids, and palmitaldehyde with 41 to 85% depletion. Uptake of three lipids was found in *P. marinivivus* and *L. algarum*. *L. algarum* also had a wide range of nucleotide and nucleosides utilization as indicated by 41–76% depletion, and *L. marina* had high uptake of adenine, adenosine, and S-methyl-5′-thioadenosine. In contrast, *P. marinivivus* showed no depletion of nucleotide or nucleosides greater than 20%. Carbohydrates such as D-mannitol were removed by *P. marinivivus*, but their depletion was less than 20% (Additional file 8: Table S7). The metabolite analysis revealed that the three isolates could utilize nitrogen compounds and lipids, but not sugars, as preferred carbon sources. *P. marinivivus* displayed preferential consumption of amino acids and dipeptides, while *L. algarum* preferred amino acids, dipeptides, nucleotides, and nucleosides. Preference for nucleotides and nucleosides was also shown by *L. marina*.

**Fig. 7:**
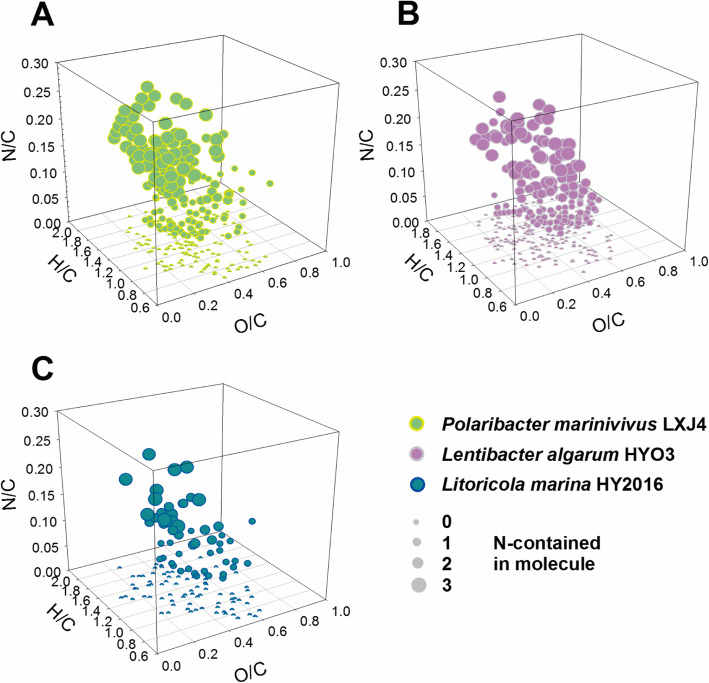
Space Van Krevelen diagram of uptake features in metabolites during growth between three isolates. Each molecule identified by FT-ICR-MS are shown as one sphere in space Van Krevelen diagrams representing the complete depleted substrates in medium (suggesting 100% uptake by bacteria) during growth, respectively cultivated with *P. marinivivus* LXJ4 (**a**), *L. algarum* HYO3 (**b**), and *L. marina* HY2016 (**c**). The elemental characteristics of molecules are shown by O/C, H/C, and N/C ratios in the *x*, *y*, and *z* axes. The CHON-formulas have the larger size with the increasing N-contained numbers in molecule

Further, Fourier-transform ion cyclotron resonance mass spectrometry (FT-ICR-MS) was used to characterize the dynamics of molecular components during growth. A total of 3089 different molecular formulas were detected within the mass range of 205–668 Da (Additional file 9: Table S8). Of these, 37% of the molecules contained at least one nitrogen atom. Figure 7 shows the complete depletion (100% uptake) of molecules during growth of the three isolates (289, 335, and 135 molecules respectively in *P. marinivivus*, *L. algarum*, and *L. marina*). However, the nitrogen-containing molecules accounted for 58% (167 formulas), 47% (158 formulas), and 39% (53 formulas) of the depletion, respectively (Fig. 7). This indicated the nitrogen-containing molecules from the bloom were preferentially taken up by the isolates.

### Implications for roles of opportunists in the bloom

From the combined perspective of genomic, proteomic, and metabonomic in the study, DON was crucial for the dominant bacteria to have a competitive advantage in blooms. Previous studies generally found that *Flavobacteriales* and *Roseobacter* clade prefer to utilize algal-derived DOM [4]. Within these two clades, *Flavobacteriales* prefer to utilize high molecular DOM, including polysaccharides, proteins, and fatty acids [4, 20, 56]. While simple sugars, nucleotides, amino acids, and other low molecular substances are preferentially used by the *Roseobacter* clade [4, 19]. Therefore, they coexist in bloom niches that differ by their DOM preferences [15, 19]. Our results indicated a similar DOM preference strategy for the three types of bacteria in our study. *P. marinivivus* expressed more peptidases that utilized the abundant dipeptide substances. *L. algarum* possessed a greater capacity in transporting and metabolizing amino acids. While *L. marina* preferred to utilize nucleotides in addition to amino acids. More interestingly, the three types of bacteria streamlined their genes by elimination of redundancy such as GH and PUL involved in carbohydrate metabolism, but retaining genes in DON transportation and utilization. Similarly, SAR11 has a strong ability to metabolize DON [57] and is also dominant in blooms (Fig. 2). These retained genes were highly expressed during the bloom, suggesting that highly efficient utilization of DON enables these streamlined bacteria to dominate the microbial community of blooms.

Nitrogen cycling plays a significant role in regulating the interactions between microbes and algae during blooms [14]. Inorganic nitrogen, including nitrate, nitrite, and ammonium, can be obtained from the ambient environment and DON mineralization. However, microbes are less able to compete with the high biomass phytoplankton for inorganic nitrogen in blooms [58], thus resulting in these microbes utilizing the phytoplankton-induced DON for their needs. We found the center sites of bloom, C(I) and C(II), were depleted in ammonium (Table 1), as the explosion of algae required a large amount of inorganic nitrogen. Nitrate concentrations during the stationary phase of the bloom were higher than the half-saturation constant for the uptake of nitrate by *A. sanguinea* (3.8 μmol l^−1^) [59], indicated that phytoplankton cells were not in a state of nitrogen-limitation. The ammonium was basically depleted due to the preference for this compound by dinoflagellates [60]. However, our three isolates had only minimal expression of the transporters or reductases for nitrate and nitrite (Additional file 7: Table S6) to acquire inorganic nitrogen. *P. marinivivus* and *L. algarum* even released some additional ammonium into the medium (Fig. 4b), potentially extending the duration of the bloom. It was speculated that the bloom environment which was DOM-replete, but ammonium-depleted, may be an important factor in selecting bacteria with high protein expression in DON utilization.

## Conclusions

In this study, we combined field and laboratory experiments to assess the bioavailability of dinoflagellate bloom-derived organic matter to opportunistic bacteria. The cultivated representatives of these opportunists (*P. marinivivus*, *L. algarum*, and *L. marina*) all exhibited streamlined genomes, featuring high protein expression in the uptake and metabolism of nitrogen-containing DOM metabolites, revealing their adaptive strategy to successfully exploit bloom-derived organic matter. Replete DOM but depleted ammonium during stationary phase of bloom may have shaped the special lifestyles of these opportunistic bacteria.

## Methods

### Sampling collection and environmental measurements

Chlorophyll a and dissolved oxygen were observed daily via a YSI EMM2000 water quality automatic monitoring buoy system with an EXO2 sensor in Wuyuan Bay, Xiamen, China (24.58° N, 118.18° E), during February to March 2016. A dinoflagellate bloom was caused by *Akashiwo sanguinea*, and samples were collected during the early- (stage I) and mid- (stage II) stationary phase of the bloom (11 days apart) at central (C) (24.59° N, 118.16° E) and peripheral (P) (24.54° N, 118.20° E) sites about 1–3 km offshore the Xiamen City coast. Temperature, salinity, and pH were measured continuously using a YSI 6600 multi-parameter meter fitted in the under way measurement system [61]. Nitrate, nitrite, phosphate, and silicate were analyzed according to standard colorimetric methods with a Technicon AA3 Auto-Analyzer (Bran-Luebbe, Germany) [62]. Ammonium was analyzed using the indophenol blue spectrophotometric method [63]. Prior to DOC measurement, water samples were acidified to pH 2 with H_3_PO_4_ and then measured using high-temperature catalytic combustion in a Shimadzu TOC-VCPH/CPN Total Organic Carbon Analyzer equipped with an ASI-V autosampler and a TNM-1 module [64]. Milli-Q water was used for a blank subtraction, and reference deep seawater (provided by the D. Hansell laboratory, University of Miami, Miami, FL, USA) served as an additional control. Total prokaryotic abundance was measured with a BD Accuri C6 cytometer stained with SYBR Green I [65–67]. The seawater samples were collected by a cap-type water extractor (Version QCCC-5) from the top 1 m of the water column, and pre-filtered with a 200-μm nylon screen. Algal cells were collected with a GF/F filter for intracellular DMSP concentration analysis and the filtrate was retained for MS analysis of metabolites. The remaining seawater was separated using polycarbonate filters into fractions dominated by free-living bacteria (3–0.2 μm size) and particle-associated bacteria (200–3 μm size) for nucleic acid sequencing [17].

### Extraction and MS analysis of metabolites from bloom

Solid-phase extraction (SPE) with styrene divinyl benzene polymer type sorbents (Varian PPL) for bloom DOM enrichment using the filtrate was performed using standard methods [68]. The concentrated solutions for LC-MS/MS analyses were studied using an Ultra-High-Performance Liquid Chromatography (UHPLC) system (1290, Agilent Technologies) with a UHPLC BEH Amide column (1.7 μm, 2.1 × 100 mm, Waters) coupled to Triple TOF 6600 (Q-TOF, AB Sciex). The column was operated at a flow rate of 0.5 ml min^−1^ with a 2-μl injection volume, equilibrated with 95% buffer B (acetonitrile) mixed with 5% buffer A (25 mM NH_4_OAc and 25 mM NH_4_OH in water, pH 9.75) for 7 min, diluting buffer B down to 65% with buffer A over 1 min, down to 40% buffer B over 1.1 min, and a return to 95% buffer B over 2.9 min. The Triple TOF mass spectrometer was used for its ability to acquire MS/MS spectra on an information-dependent basis (IDA) during LC/MS analyses. In this mode, the acquisition software (Analyst TF 1.7, AB Sciex) continuously evaluated the full scan survey MS data. In each cycle, 12 precursor ions, with intensities greater than 100, were chosen for fragmentation at collision energy (CE) of 30 V (15 MS/MS events with product-ion accumulation time of 50 ms each) [69]. Electrospray ion source (ESI) source modified conditions were as follows: ion source gas 1 as 60 Psi, ion source gas 2 as 60 Psi, curtain gas as 35 Psi, source temperature 650 °C, and ion spray voltage floating (ISVF) 5000 V or − 4000 V in positive or negative modes, respectively [70]. The MS data were converted to mzXML format using ProteoWizard [71, 72] and processed by R package XCMS (Version 3.2). The preprocessing results generated a data matrix that consisted of the retention time (RT), mass-to-charge ratio (m/z) values, and peak intensity. R package CAMERA was used for peak annotation after XCMS data processing. An in-house MS/MS library (> 1400 metabolites) was used for metabolite identification (matching major MS/MS fragments and > 0.6 MS/MS score). For the metabolites identified in our study (Additional file 4: Table S3), we provide a classification of metabolite identification recommended by the Human Metabolome Database (HMDB, http://www.hmdb.ca/). Mantel Test with the Pearson’s correlation coefficient was performed to determine statistically significant correlation via PAleontological Statistics (PAST, Version 3.25).

The filters with algal cell samples were extracted by acetonitrile: methanol: water (v/v/v 2:2:1) and centrifuged at 12,000 r.p.m. and 4 °C for 15 min. The supernatant for intracellular DMSP concentration were analyzed using an Agilent 1290 Infinity II series UHPLC System (Agilent Technologies), equipped with a UPLC BEH Amide column (1.7 μm, 2.1 × 100 mm, Waters) coupled to Agilent 6460 triple quadrupole mass spectrometer with an AJS electrospray ionization (AJS-ESI) interface. The MRM parameters for the targeted analyte were optimized, by injecting the standard solutions of the individual analytes directly into the API source of the mass spectrometer. Agilent MassHunter Work Station Software (B.08.00, Agilent Technologies) was employed for MRM data acquisition and processing. Calibration curves performed by the standard of DMSP (Santa, USA) were generated by plotting peak area against concentration.

### Nucleic acid sequencing and analysis

Environmental bacterial DNA from polycarbonate filters was extracted using a Fast DNA SPIN Kit for Soil (Mpbio, USA). To generate 16S (bacteria) ribosomal RNA gene libraries for tag-sequencing, we constructed short-read Illumina and full-length Pacific Biosciences (PacBio) libraries to provide high-resolution analysis of the microbial communities [73, 74]. For the Illumina library, the sequencing primer set 16S-343F: TACGGRAGGCAGCAG, and 16S-798R: AGGGTATCTAATCCT was chosen for PCR conditions. The PCR products were purified with AMPure XT beads (Danvers, USA) and mixed into a pool according to the sequencing volume requirement after fluorescence quantification (Qubit 2.0 Fluorometer) using a Qubit dsDNA HS Assay Kit (Invitrogen, USA). The 16S rRNA genes were sequenced using the Illumina MiSeq™ platform (USA) as described elsewhere [75]. For the PacBio library, broad PCR conditions were achieved using the gene-specific primer 16S-27F: AGAGTTTGATCATGGCTCAG, and 16S-1492R: ACGGYTACCTTGTTACGACTT. The PCR products were purified using a QIAquick PCR Purification Kit (Qiagen, Germany) and mixed into a pool according to the sequencing volume requirement after fluorescence quantification (Qubit 2.0 Fluorometer) using a Qubit dsDNA HS Assay Kit (Invitrogen, USA). Sequencing libraries were generated using a PacBio® SMRTbell™ Template Prep Kit (PacBio, USA). Pooled amplicons were purified with the AMPure® Beads (Beckman, USA) and the SMRTbell templates sequenced on the PacBio RS II platform (USA) referred previously [74]. The ReadsOfInsert protocol in SMRT Analysis v2.3 (PacBio, USA) was used for data quality control for the PacBio 16S rRNA genes. Filtering, mapping, chimera detection, and clustering were performed using a set of Mothur tools (unique.seqs, align.seqs, chimera.uchime v4.2, classify.seqs, dist.seqs, cluster, mapping with silva v123 aligned ribosome sequence database) with default parameters [76, 77], and sequence reads were clustered into operational taxonomic units at the 97% similarity level. At the species level, the PacBio 16S RNA sequences were mapped with EzBioCloud 16S Database (Version 2018.05, download from https://www.ezbiocloud.net) using BLAST/ BLAST analyses and defined at 99% similarity level of the 16S rRNA genes to species. Illumina pair-end reads were mapped to the assembled contigs to improve the accuracy of genome sequences. To assess whether there were significant differences in the genera of the microbial communities. Principal component analysis between the relative abundance of genera indicated the variation of microbial community composition in bloom. ANOSIMS analysis performed in PAST (Version 3.25) was applied to estimate the factors driving statistically significant differences (stages, sites, or strategies) between the microbial communities during bloom.

### Bacterial isolation, growth, and culture condition

Bacterial isolates (detailed in Additional file 3: Table S2) were obtained by serial dilution using two mediums. The mediums were prepared by filtered, natural autochthonous seawater collected during bloom or a marine 2216E Agar medium (BD, USA). Isolates of *Polaribacter marinivivus* LXJ4, *Lentibacter algarum* HYO3, and *Litoricola marina* HY2016 were routinely grown in filtered sterile autochthonous seawater from the bloom, in 50-ml Erlenmeyer flasks incubated at 16 °C with shaking (160 r.p.m.). Cells were collected and washed thrice with autoclaved ASW solution [78] to eliminate nutrients before inoculation. Isolate cell abundance was monitored by a flow cytometer (BD FACScan) with independent biological triplicates. Cells were collected by centrifugation (3500×*g* for 5 min at 4 °C) during the exponential phase and at the end of the stationary phase and washed three times using 0.01 mol l^−1^ sterile PBS (pH 7.2–7.4) for proteomics analysis. The filtered solution from the end of the stationary growth phase and uninoculated starting medium were collected for analysis of metabolite uptake and the concentration changes of DOC and ammonium.

### Whole-genome sequencing and analysis

Whole-genome sequencing was performed using a hybrid approach [79], combining Illumina short-read data (Illumina, USA) with PacBio long-read data (PacBio, USA). SMRTbell DNA template libraries were constructed and SMRT sequencing was performed on the Pacific Biosciences RSII sequencer (PacBio, USA). The genome sequences were de novo assembled by the HGAP2 program in the SMRT analysis server (v2.3), and Illumina pair-end reads were mapped to the assembled contigs to improve the accuracy of genome sequences [80]. The final assembled genomes were automatically annotated and analyzed through the Joint Genome Institute Integrated Microbial Genomics (IMG) site (http://img.jgi.doe.gov). *Lentibacter algarum* HYO3 was referenced using the closest match, *L. algarum* DSM 24677 (IMG ID 2693429861). Genomic information of reference strains belonging to the *Flavobacteriia*, *Roseobacter*, and *Oceanospirillales* were downloaded from the IMG site. Peptidase genes were annotated using the MEROPS peptidases database [81]. The candidates were manually examined in terms of similarity (*E*-value cutoff 1e− 10) to MEROPS proteins and the presence of all catalytic sites. The amino acid sequences were then submitted to the CAZyme Annotation Toolkit (http://mothra.ornl.gov/cgi-bin/cat/cat.cgi) for sequence-based annotation [82, 83], with an *E*-value of 1e− 40, as well as Pfam-based annotation with an *e*-value of 0.00001. The manually selected sequences of DON membrane transporter were blasted (*E*-value cutoff 1e− 5) with the reference strains annotated using the Transporter Classification Database (http://www.tcdb.org) [84]. The results were then further checked manually.

### Preparation of cellular proteomes for nano-LC-MS/MS and data analysis

Shotgun proteomic analysis of cellular extracts of isolates grown in autochthonous seawater medium was carried out to elucidate the metabolic mechanisms utilizing the bloom DOM. Cell pellets were collected and lysed by SDT buffer (4% SDS, 100 mM DTT, 150 mM Tris-HCl pH 8.0) and the protein concentration was quantified with the BCA Protein Assay Kit (Bio-Rad, USA) [85]. The protein suspensions were digested with 4 μg trypsin (Promega) in 40 μl 25 mM NH_4_HCO_3_ buffer overnight at 37 °C as previously described [86] and the resulting peptides were collected as a filtrate. The peptides were analyzed by nano-LC-MS/MS with a reverse-phase trap column (Thermo Scientific Acclaim PepMap100, 100 μm×2 cm, nanoViper C18) connected to the C18-reversed-phase analytical column (Thermo Scientific Easy Column, 10 cm long, 75-μm inner diameter, 3-μm resin) in buffer A (0.1% Formic acid) and separated with a linear gradient of buffer B (84% acetonitrile and 0.1% formic acid) at a flow rate of 300 nl min^−1^ controlled by IntelliFlow technology. The 4 h linear gradient was 0–55% buffer B for 220 min and 55–100% buffer B for 8 min and held in 100% buffer B for 12 min. LC-MS/MS analysis was performed on a Q Exactive mass spectrometer (Thermo Scientific) that was coupled to Easy nLC (Proxeon Biosystems, now Thermo Fisher Scientific) for 240 min. The mass spectrometer was operated in a positive ion mode. MS data were acquired using a data-dependent top 10 method dynamically choosing the most abundant precursor ions from the survey scan (300–1800 m/z) for HCD fragmentation. Survey scans were acquired at a resolution of 70,000 at m/z 200, resolution for HCD spectra was set to 17,500 at m/z 200, and isolation width was 2 m/z. Normalized collision energy was 30 eV, and the underfill ratio, which specifies the minimum percentage of the target value likely to reach at maximum fill time, was defined as 0.1%. The MS data were analyzed using MaxQuant software version 1.5.3.17 (Max Planck Institute of Biochemistry in Martinsried, Germany) [87].

### Uptake of DOM components analysis

The variation of DOM components during growth of isolates was assessed by LC-MS/MS and FT-ICR-MS analysis. At the end of the stationary growth phase, the culturing solution was filtered through a GF75 filter and the filtrate collected in triplicates. Uninoculated medium was used as the control.

The filtrate for SPE enrichment and LC-MS/MS analyses were discussed in detail in “Extraction and MS analysis of metabolites from bloom”. Metabolites that were identified as having a 20% decrease in peak intensity from the uninoculated medium and shown to be statistically significant (*p* value < 0.05) by *T*-test analysis were considered to be taken up by the isolate (details in Additional file 8: Table S7).

DOM enrichment samples were adjusted to yield 25 mmol l^−1^ DOC and analyzed using a Bruker Apex Ultra FT-ICR-MS equipped with a 9.4 T superconducting magnet. Sample solutions were infused via an Apollo II ESI at 250 μl h^−1^ with a syringe pump. Typical operating conditions for negative ESI were as follows: spray shield voltage 3.0 kV, capillary column introduce voltage 3.5 kV, and capillary column end voltage − 320 V. The mass range was set to m/z 200–800. The 2M word size was selected for the time-domain signal acquisition. A number of 128 time-domain signals were co-added to enhance the signal-to-noise ratio and dynamic range. The magnitude threshold for the peak assignment was set to a signal-to-noise ratio of ≥ 4, with a standard deviation < 0.2 ppm for the MS. The FT-ICR-MS was calibrated using a known homologous series of the Suwannee River natural organic matter sample (obtained from IHSS, USA), which contained a relatively high abundance of oxygen-containing compounds.

## Supplementary Information

**Additional file 1: Figure S1.** Phytoplankton bloom observed in the ocean off the coast of Xiamen. **Figure S2.** Growth of three isolates. **Figure S4.** Protein expression rank of LFQ intensity during growth in three isolates. **Figure S4.** Genetic organization of the predicted laminarin and alginate PULs of P. marinivivus LXJ4. **Figure S5.** Analysis of dimethylsulfoniopropionate (DMSP) via targeted LC MS/MS.

**Additional file 2: Table S1.** Metabolites identified in bloom.

**Additional file 3: Table S2.** Dynamics of bacterial community composition.

**Additional file 4: Table S3.** Cultivable bacteria isolated from the bloom.

**Additional file 5: Table S4.** Genomic information and encoded DON transporters on isolates and reference strains.

**Additional file 6: Table S5.** Summary and distributions of peptidases and CAZymes in sequenced and reference genomes.

**Additional file 7: Table S6.** Proteomics datasets generated from three isolates grown with bloom-derived DOM in mid-exponential and late stationary phases.

**Additional file 8: Table S7.** Uptake of the components of DOM by isolates via LC-MS/MS.

**Additional file 9: Table S8.** Uptake of the components of DOM via FT-ICR-MS.

## Data Availability

The 16S rRNA gene sequences of the bloom bacterial community have been deposited in the NCBI Sequence Read Archive (SRA) under BioProject accession numbers PRJNA591695 with Illumina Miseq platform and PRJNA595399 with Pacific SMRT platform. All isolates with the 16S RNA genes from bloom have been deposited in the NCBI under GenBank accession numbers KX755349-KX755376, KX784511-KX784546, and MN746114-MN746265. The complete genome sequences of *Polaribacter marinivivus* LXJ4 and *Litoricola marina* HY2016 have been deposited in the Joint Genome Institute IMG/ER website under the genome IDs 2773857722 and 2773857723, respectively. Proteomics data files of *P. marinivivus* LXJ4, *L. algarum* HYO3, and *L. marina* HY2016, including raw files (raw) and search files (txt), have been respectively deposited to the PRIDE/ProteomeXchange database under the accession numbers PXD016858, PXD016786, and PXD016572.

## Abbreviations

DOM: Dissolved organic matter
DOC: Dissolved organic carbon
Chl *a*: Chlorophyll a
DO: Dissolved oxygen
ANOSIMS: Analysis of similarities
CRISPR: Clustered regularly interspaced short palindromic repeats
GH: Glycoside hydrolase
ABC: The ATP-binding cassette
COG: Clusters of orthologous gene
TBDT: TonB-dependent
PUL: Polysaccharide utilization loci
DMSP: Dimethylsulfoniopropionate
FT-ICR-MS: Fourier-transform ion cyclotron resonance mass spectrometry
SPE: Solid-phase extraction
UHPLC: Ultra-High-Performance Liquid Chromatography
ESI: Electrospray ion source
PacBio: Pacific Biosciences
PAST: PAleontological Statistics
IMG: Integrated Microbial Genomics
SRA: Sequence Read Archive
Temp: Temperature
DON: Dissolved organic nitrogen
TPA: Total prokaryotic abundance
N.D.: Not detectable
