# Supplementary material for: Opportunistic bacteria with reduced genomes are effective competitors for organic nitrogen compounds in coastal dinoflagellate blooms
Source: Microbiome. 2021 Mar 24;9:71. doi: 10.1186/s40168-021-01022-z (PMC7992965; doi:10.1186/s40168-021-01022-z)
Supplement: Supplementary file 2 — Additional file 1: Figure S1. Phytoplankton bloom observed in the ocean off the coast of Xiamen. Figure S2. Growth of three isolates. Figure S4. Protein expression rank of LFQ intensity during growth in three isolates. Figure S4. Genetic organization of the predicted laminarin and alginate PULs of P. marinivivus LXJ4. Figure S5. Analysis of dimethylsulfoniopropionate (DMSP) via targeted LC MS/MS. [file 40168_2021_1022_MOESM2_ESM.docx]

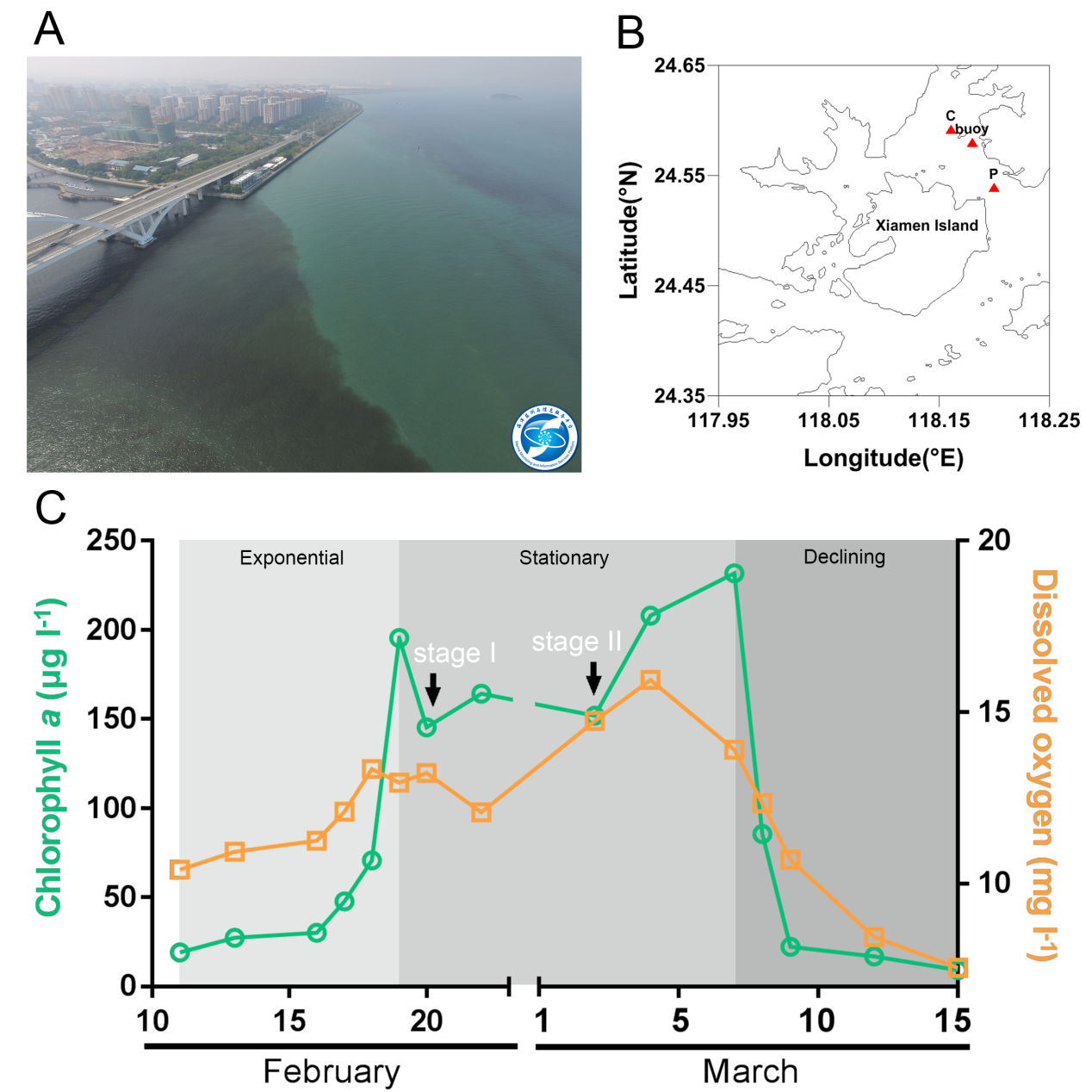


Figure S1. Phytoplankton bloom observed in the ocean off the coast of Xiamen. Partial aerial photo of the algal bloom taken by a drone (A). The sampling sites included the center (C), periphery (P), and buoy areas of the algal bloom (B). Chlorophyll a (circles with a green line) and dissolved oxygen (squares with a black line) observed in the exponential to the declining phase of the bloom between February and March 2016. Samples were collected in early- (stage I) and mid- (stage II) stationary phase (pointed out with arrows (C)).


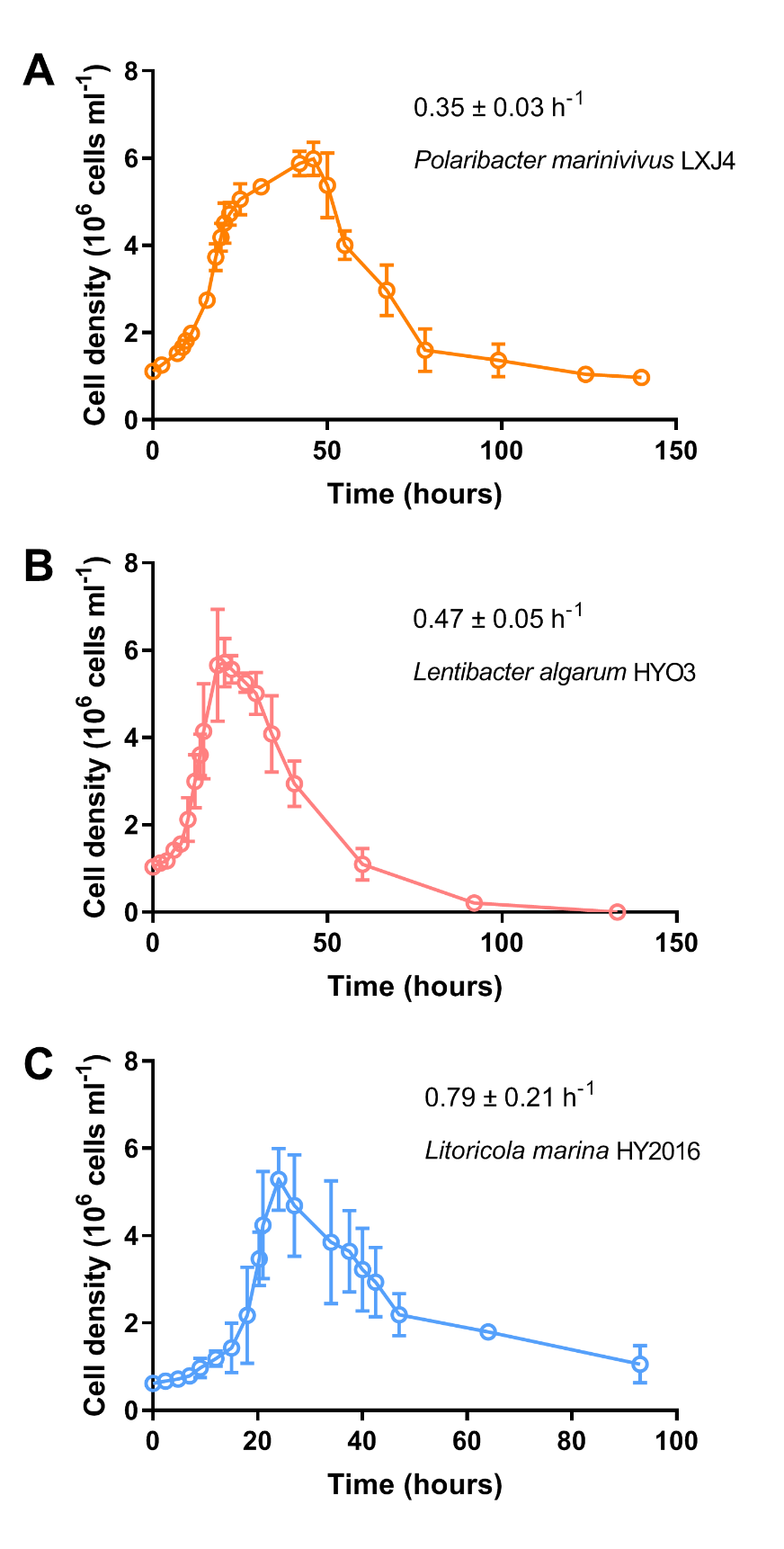


Figure S2. Growth of three isolates. *P. marinivivus* LXJ4 (A), *L. algarum* HYO3 (B), and *L. marina* HY2016 (C) cultured in autochthonous-filtered seawater medium from bloom with the growth rate attached to the side of the curve.


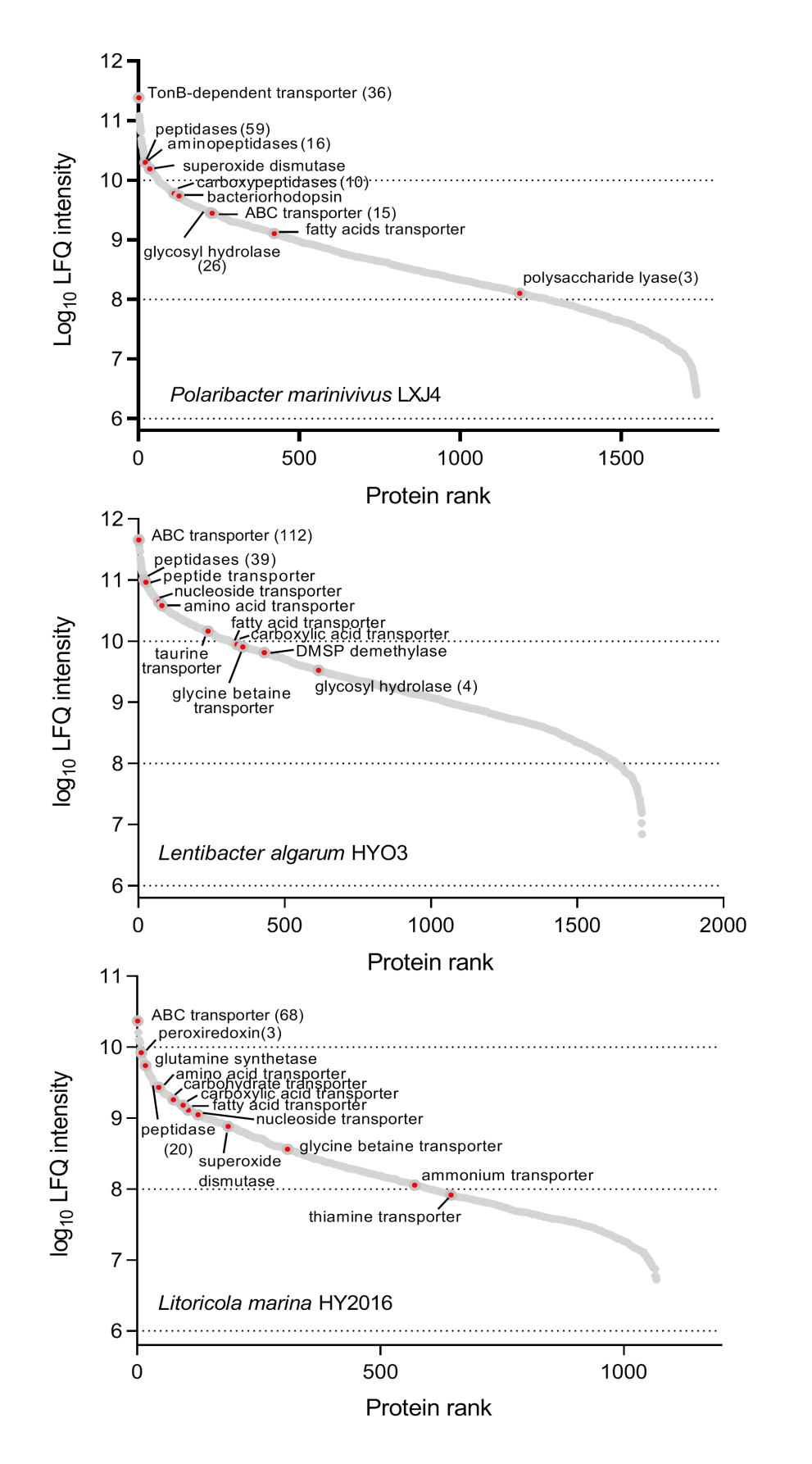


**Figure S3.  Protein expression rank of LFQ intensity during growth in three isolates.** The x axis indicates the rank of identified protein, and the y axis indicates the median of LFQ intensity in a log10 scale. The proteins we described are highlighted with red points, and numbers in brackets show the expression of homologous proteins in the incubation.


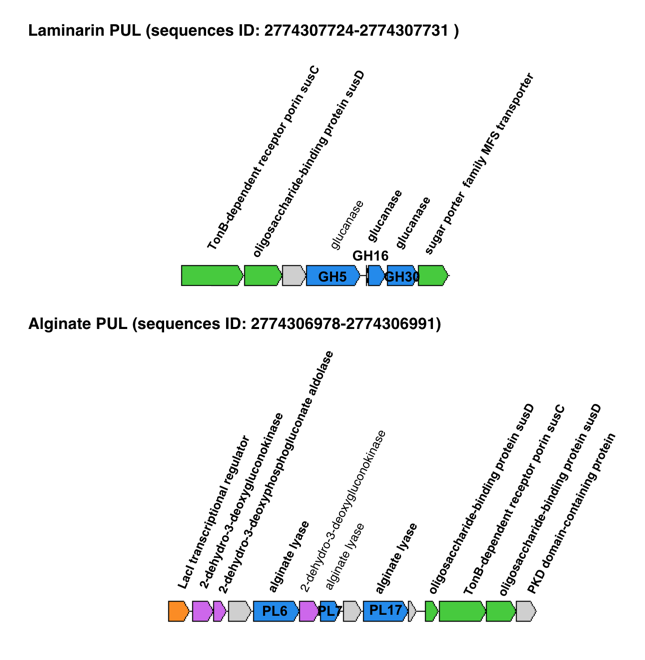


Figure S4.  Genetic organization of the predicted laminarin and alginate PULs of *P. marinivivus* LXJ4. The expressed proteins were highlighted by bold texts. The functions of proteins are color-coded: blue, green, membrane proteins involved in binding/transport; purple, other enzymes; orange, regulation factor; gray, unknown function. The sequence ID of the gene cluster in the genome is shown in a bracket (https://img.jgi.doe.gov).


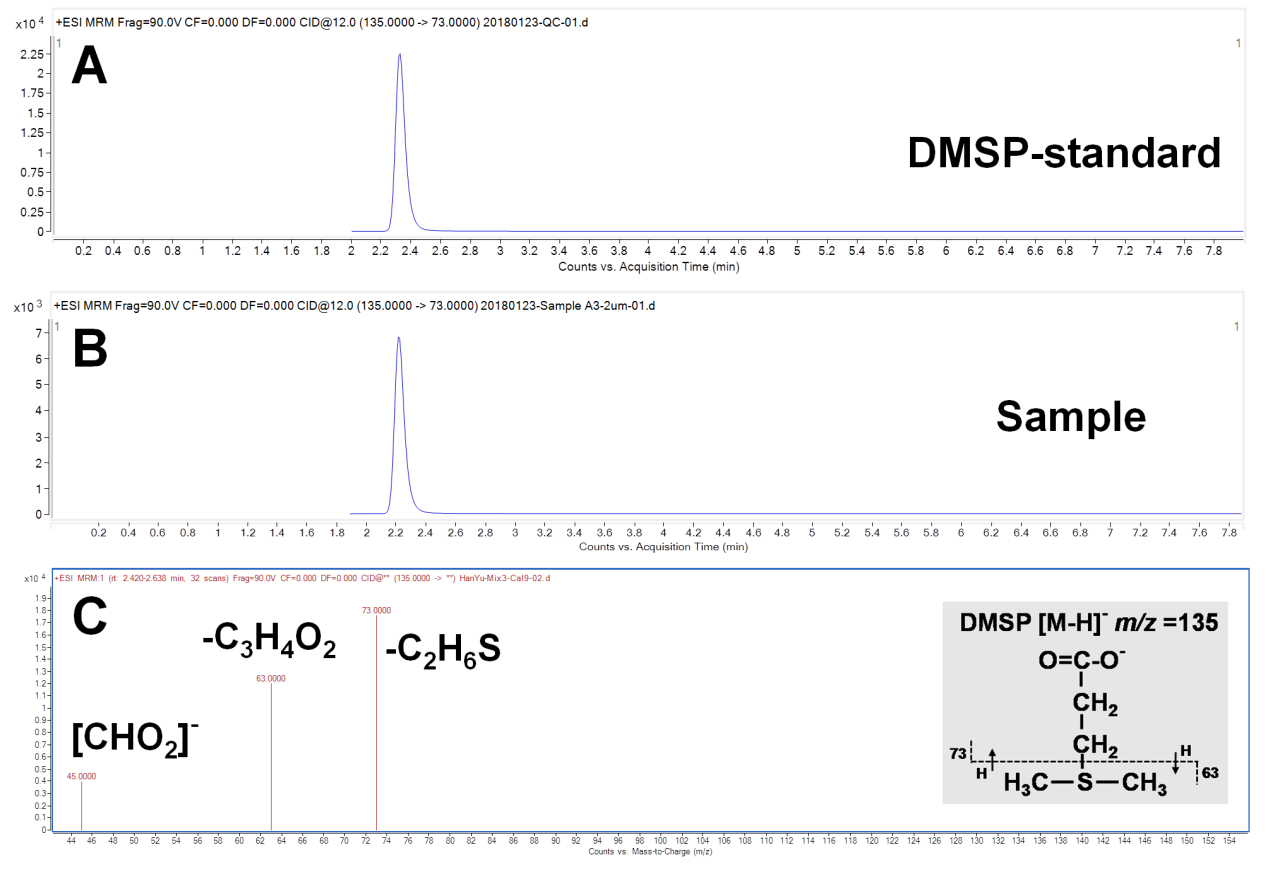


Figure S5. Analysis of dimethylsulfoniopropionate (DMSP) via targeted LC-MS/MS. The same retention time on DMSP standard solution (A) and bloom sample (B) with the MS/MS characteristics of DMSP (C).
